# The Relative Importance of Training and Social Support for Runners’ Performance: A Cross-Sectional Study

**DOI:** 10.1186/s40798-023-00557-9

**Published:** 2023-02-23

**Authors:** Mabliny Thuany, Douglas Vieira, Henrique de Paula, Pantelis T. Nikolaidis, Volker Scheer, Katja Weiss, Ivan Cuk, Beat Knechtle, Thayse Natacha Gomes

**Affiliations:** 1 Centre of Research, Education, Innovation and Intervention in Sport (CIFI2D), Faculty of Sports, University of Porto, 4200-450 Porto, Portugal; 2 Post-Graduation Program of Physical Education, Federal University of Sergipe, São Cristóvão, SE Brazil; 3 School of Health and Caring Sciences, University of West Attica, 12243 Athens, Greece; 4 Ultra Sports Science Foundation, Pierre-Bénite, France; 5 Institute of Primary Care, University of Zurich, 8091 Zurich, Switzerland; 6 Faculty of Physical Education and Sports Management, Singidunum University, Belgrade, Serbia; 7 Medbase St. Gallen Am Vadianplatz, Vadianstrasse 26, 9001 St. Gallen, Switzerland; 8 Department of Physical Education and Sports Science, University of Limerick, Limerick, Ireland; 9 Physical Activity for Health Cluster, Health Research Institute, University of Limerick, Limerick, Ireland

**Keywords:** Complex systems, Endurance, Exercise intensity, Network model, Behavior

## Abstract

**Background:**

Running participation/performance is a complex system. Understanding the variables associated with these behaviors may help to increase population physical activity and improve performance. This study aimed to investigate social and training variables important for running performance using a network approach.

**Methods:**

This cross-sectional study sampled 1151 non-professional Brazilian runners of both sexes (women, 38.2%; men, 61.7%). A questionnaire was available for eligible participants using an online platform, and information regarding training (volume and running pace) and social variables (participation in a running event, participation in a running group, influence on running, runners in the family, and childhood sport) related to runners’ performance was obtained. The Chi-square test and network model were estimated by sex.

**Results:**

Training characteristics tend to be clustered. For both sexes, the training volume node presented the highest expected influence in the network (1.69 for women and 2.16 for men). Centrality indicators of social variables show that sports childhood participation and the presence of runners in the family were the most important nodes of network connection for women and men, respectively.

**Conclusion:**

Based on these findings, it was concluded that sports participation during childhood and the practice of running by other family members were important factors to connect variables in the network. These findings have practical applications for health policymakers, highlighting the need to develop educational programs to increase sports participation during childhood and within families.

## Key points

- Sports participation during childhood was the most important social variable in female runners’ performance;
- Running practice in the family was related to male runners’ performance;
- Among social and training variables, volume for the week presented the strongest expected influence for runners of both sexes.

## Background

Physical inactivity is one of the leading public health concerns at the world level [1, 2]. The World Health Organization Global Action Plan on Physical Activity (2018–2030) includes promoting physical, mental health and well-being to tackle these concerns [3]. Mass events (i.e., running, walking) are considered tools to achieve these goals [4], considering the health-related outcomes (e.g., decrease in mortality risk, increase in longevity), psychological benefits (e.g., well-being, resilience, coping), and the low cost of practice compared to other physical activities performed in structured space [5].

Running is considered a social phenomenon [6, 7]. Race events participation and running performance are the results of a complex interaction between athlete (e.g., economic support, training, motivation, physiological development), environment (e.g., race event available, social security to training), and task (e.g., motor efficiency to running) constraints [8, 9]. These aspects highlight running as a complex system; however, few studies consider this theoretical framework to deeply understand this phenomenon [9, 10].

Considering running behaviors as a complex system includes identifying the existence of simple rules for the emergence of different behavior. In complex systems, simple rules are the basic actions responsible for triggering emergent behavior patterns [11]. Through social support and social comparison, runners tend to be more committed in training, increasing weekly volume, frequency, and performance. A global social network aiming to understand the social contagious of running patterns [12] showed that active runners are positively influenced by those less actives, while both females and males influence the running practice of male friends [12]. In another way, female runners tend to be influenced by other females [12]. Similarly, training habits and runners’ goals (i.e., participating in a race) were positively related to social network support [13].

Running patterns were also studied considering the role of family support, to belong a running club, or having a coach [7, 14, 15]. Results showed that the presence of runners in family was a pivotal event to start to run and to maintain people engaged in long-term running program [15]. However, the role of the social support for running engagement tends to be different between sexes, especially considering inequalities for physical activity in adults [16]. Barriers related to perception of security, double work journey and harassment [17] strongly and negatively influence female training, through the changing of route, decrease the time of the run, and stop running alone [17].

Considering the potential of running to increase physical activity levels, to understand the main variables related to the training engagement is important to provide advances in physical activity promotion. This study aims to investigate social and training variables critical to running performance using a network approach. The network analysis is an approach used to understand complex behaviors, through the interaction between variables of different levels [18]. We hypothesized that training variables present the highest importance for runners’ performance [19], whereas social variables, such as having a runner in the family, can present essential social support, especially for women, considering the barriers for physical activities in outdoor spaces.

## Methods

### Design and Sample

A total of 1242 subjects were recruited from the first wave of the InTrack project [20], a cross-sectional study developed in all Brazilian federative units. To be included, runners were invited to answer an online questionnaire (https://forms.gle/WaiKTstu8WQjg82B8), self-classify as a runner, and accept the study participation. Exclusion criteria included not answering all the mandatory questions from the provided questionnaire (i.e., anthropometric variables, running pace, and training volume) and age younger than 18.

### Procedures for Data Collection

The questionnaire “Profile characterization and associated factors for runner’s performance” was developed and previously validated [21]. The questionnaire was available for eligible participants using an online platform (Google forms) between September 2019 and March 2020. This online strategy was chosen to cover all the Brazilian states, although it was not meant to obtain representative information for all Brazilian runners. For the present study, the following variables were used:

### Individual Characteristics

Sex (female, male), age (years), body height (m), and body weight (kg) were self-reported. Body mass index (BMI) was computed by the standard formula [weight (kg)/height (m^2^)].

### Training Characteristics

#### Running Pace

The time (minutes) spent to cover one kilometer was used as the performance index. Participants were also asked to provide information on their running pace in their preferred distance (5 km, 10 km, half-marathon, and marathon). For the present study, we considered running pace in seconds/kilometer (s/km).

#### Training Experience

The time in which the participant is committed in running training was estimated. The training experience was considered in years, dichotomized as ‘until 1 year’ and ‘higher than 1 year’.

#### Training Frequency

Runners were asked to state the number of weekly training sessions they usually complete (2–7 sessions/week). The variable was considered in “≤ 3 training/week” or “> 3 training/week”.

#### Training Volume/Week

Runners were asked to provide information about the average total distance (km) they usually cover during their weekly training sessions.

### Social Variables

#### Participation in a Running Event

Runners were asked if they had participated in an official event in the last 12 months (“yes” or “no”).

#### Participation in a Running Group

Runners were asked if they were officially participating in a running team or group (“yes” or “no”).

#### Running Because was Influenced

Runners were asked if they have been influenced by a family member or friend to start running (“yes” or “no”).

#### Runners in the Family

Runners were asked about other family members practicing running. Answers were dichotomized into “yes” or “no”.

#### Childhood Sport

Runners were asked about their sports participation during childhood. Answers were dichotomized into “yes” or “no”.

### Statistical Analysis

Mean and standard deviation (SD) and frequencies (percentages) were used to describe participants' information. Normality was tested from the Kolmogorov–Smirnov test. The Mann–Whitney U test was used to verify differences between both sexes' demographics (i.e., age, BMI) and training variables (i.e., volume and running pace). Absolute differences (Δ) were calculated to present effect size. The chi-square test (*χ*^2^) was used to verify the association between sex (female; male) and social variables (participation in a running event; participation in a running group; influence to running; runners in the family; childhood sport).

Following, network analysis was performed to identify the complex interaction between training characteristics, social variables, and running performance. Network analysis is an approach used to identify complex patterns between variables in a system, adjusting for the interaction between all variables [18]. We used the EBICglasso estimator and presented results using a correlation matrix and centrality indicators (i.e., closeness, betweenness, and expected influence) [18]. High closeness scores might make a node more reliant on other nodes in the network because the closeness values represent the average distance between nodes. The betweenness describes how frequently a node is located on the shortest path that connects all other nodes in a network. High values may suggest that a node is an important hub connecting other nodes in the network. Variables with the highest values for expected influence are more sensitive to change [18].

Usually, network analysis is presented through the graphs that show the relationship between variables, which the nodes are variables, and the edges (links) representing the relationship between the nodes. The strength and the direction of the association are represented by the edge density and color (blue: positive association; red: negative association). We presented social and training variables clustered by colors, purple and yellow, respectively. The running pace was presented in red. Network accuracy was estimated based on 1000 resample bootstrap. Statistical analyses were performed in the JASP 0.16.1.0, considering a 95% confidence interval.

## Results

A total of 1151 recreational Brazilian runners of both sexes (women, 38.2%; men, 61.7%) with an average age of 37.9 ± 9.4 years were sampled. Descriptive information and comparative analysis are presented in Table 1. BMI was lower in women compared to men. For training variables, men presented a higher weekly training volume (Δ = 13.8 km/week), and a faster pace comparatively women (Δ = − 54 s/min). Men presented the highest frequency for practice training higher than one year (86.9%), and training frequency higher than three training/week (47.1%). For social variables, non-significant differences were shown for participation in running competitions and the presence of other runners in the family. Women presented the highest frequency of participation in a running team (80%), while 87.1% of the men reported sports practice during childhood, comparatively to 68% of the women.

**Table 1:** Descriptive information [mean ± SD or frequency (%)] and comparisons, considering both sexes

Variables	Women (*n* = 440)		Men (*n* = 711)		Mann–Whitney *U* test *p* value
	Mean	SD	Mean	SD	
Age (years)	38.0	8.5	37.9	9.9	0.555
BMI (kg/m^2^)	23.5	2.9	24.7	3.1	**< 0.001**
*Training variables*					
Training volume (km/week)	26.9	16.3	40.7	34.2	**< 0.001**
Running pace (s/km)	358.0	49.7	303.1	52.1	**< 0.001**
	Frequency	%	Frequency	%	*χ*^2^ test *p* value
Training frequency					
≤ 3 training/week	302	68.6	376	52.9	**< 0.001**
> 3 training/week	138	31.4	335	47.1	
Training experience					
Until 1 year	81	18.4	92	12.9	**0.011**
Highest 1 year	358	81.4	618	86.9	
Missing	1	0.2	1	0.1	
*Social variables*					
Running event					
No	31	7.0	49	6.9	0.921
Yes	409	93.0	662	93.1	
Running group					
No	87	19.8	207	29.1	**< 0.001**
Yes	352	80.0	504	70.9	
Missing	1	0.2			
Runners’ family					
No	285	64.8	444	62.4	0.344
Yes	152	34.3	267	37.6	
Missing	3	0.7			
Sports childhood					
No	141	32.0	90	12.7	**< 0.001**
Yes	299	68.0	619	87.1	
Missing			2	0.3	

*SD* standard deviation, *BMI* Body Mass Index, *p* < 0.001 are presented in bold

**Fig. 1:**
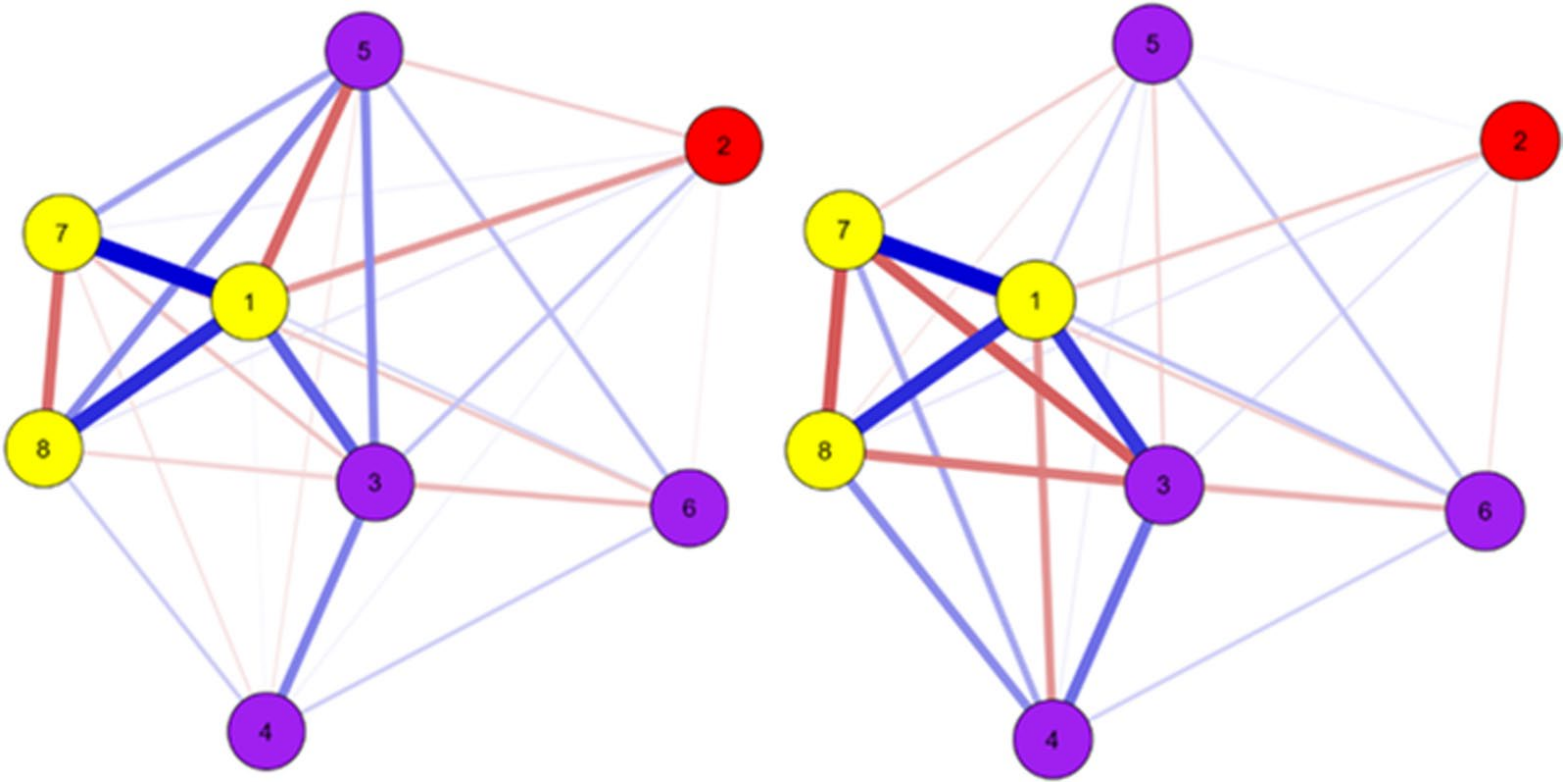
Network plot for both sexes (left panel: women; right panel: men). Legend: yellow colors—(1) training volume/week; (7) training frequency/week (> 3 train/week); (8) Running experience (> 1 year); purple colors—(3) running event (yes); (4) running group participation (yes); (5) runner in family (yes); (6) sports childhood participation (yes); red color—(2) running pace (s/km)

Figure 1 shows the network plot. Network topology is similar for both sexes, with some differences for the strength of the relationship among variables. Training characteristics tend to be clustered for both sexes (yellow nodes), while for social variables, runners in the family tend to be sparser than the other nodes (purple color).

**Fig. 2:**
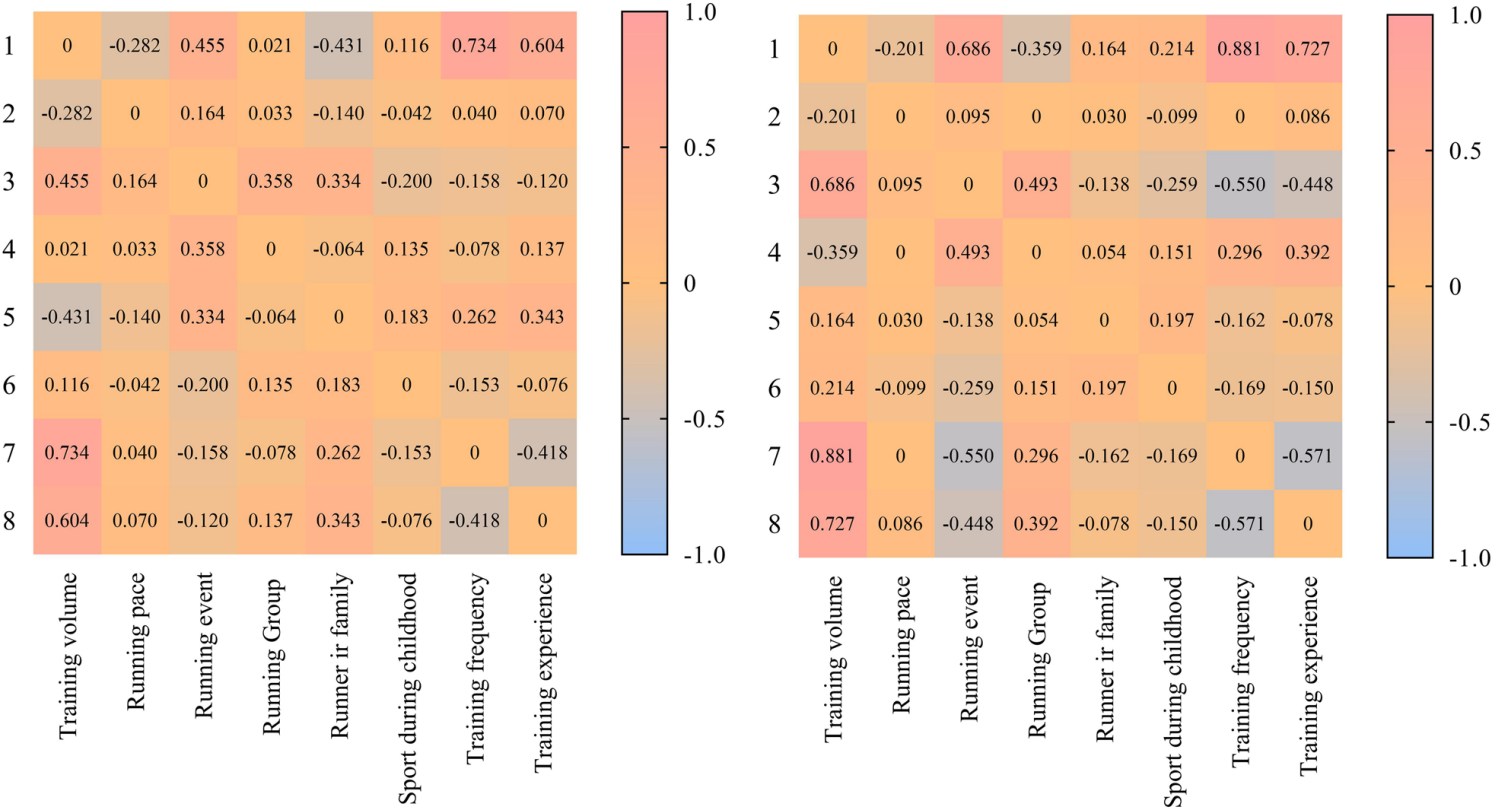
Correlation matrix for both sexes (left panel: women; right panel: men)

Figure 2 presents the correlation matrix for both sexes. Positive associations were shown between training variables in both sexes. For women, family members practicing running was positively related to training experience (*r* = 0.343), weekly training frequency (*r* = 0.262), sports participation during childhood (*r* = 0.183), and participation in a running event (*r* = 0.334). For men, family members practicing running was related to higher weekly volume (*r* = 0.164), sport during childhood (*r* = 0.197), and participation in a running group (*r* = 0.05).

Centrality indicators are presented in Table 2, for both sexes. Among training variables, weekly volume was the most important node to connect and influence other variables in the network (betweenness_women_ = 2.03; betweenness_men_: 2.413, respectively). Among social variables, sports participation during childhood and the presence of runners in the family were the most important nodes to network connection for women and men, respectively (Closeness_women_: − 1.367; Closeness_men_: − 1.256). For expected influence, sports participation during childhood and running in group were the most important variables for the network (women: − 1.102; men: 0.851).

**Table 2:** Centrality indicators result for both sexes

Variable	Women			Men		
	Betweenness	Closeness	Expected influence	Betweenness	Closeness	Expected influence
Training volume	2.034	1.442	1.696	2.413	1.339	2.161
Training frequency	− 0.560	0.445	− 0.508	− 0.435	0.712	− 0.719
Running experience	− 0.560	0.144	0.187	− 0.435	0.469	− 0.438
Running pace	− 0.560	− 1.039	− 1.369	− 0.435	− 1.271	− 0.493
Running competition	1.091	0.871	0.838	0.198	0.867	− 0.532
Running group	− 0.560	− 0.908	0.191	− 0.435	− 0.142	0.851
Runner family	− 0.324	0.413	0.067	− 0.435	− 1.256	− 0.305
Sports childhood	− 0.560	− 1.367	− 1.102	− 0.435	− 0.718	− 0.525

## Discussion

This study aimed to investigate social and training variables related with running performance using a network approach. We hypothesized that training variables present the highest importance to runners’ performance and that social variables—having a runner in the family—could have a significant effect, especially for women. The main findings point out that (a) weekly volume was the most important variable to connect and provide changes in running pace within the network; (b) sports participation during childhood for women, the presence of runners in the family, and participation in a running group for men were the most important variables in the network. These results refute our hypothesis since the presence of runners in the family was the most critical node for the network system among men.

The first result was the importance of weekly training volume for running performance. Training variables were previously related to runner’s performance in both elite and amateur subgroups [22–25]. Training is linked to a plethora of physiological changes such as the improvement of the central aerobic components, use of oxygen by the muscle, and increase in anaerobic threshold [26], through the increases in the mitochondrial content and skeletal muscle capillary density [27]. Previous studies highlighted the role of the training variables for runners’ performance, involving runners competing in 5 km to ultramarathon events [19, 28], at different performance levels (e.g., amateur and elite athletes) [25, 29, 30]. However, generalization should be done with caution, because our analysis was not controlled for distance of competition (5 km, 10 km, half-marathon, marathon- or ultramarathon), age groups [31], motivation [31–33], or psychological characteristics [23, 34], since these variables present a moderator role for training engagement.

Moving forward, for social variables, sports participation during childhood, and the presence of runners in the family, were the most important variables to connect other nodes in the network, for women and men, respectively. The role of social support in runners' participation/performance was little investigated previously [12, 15, 35]. Social support is a multifaceted concept related to reciprocal social relationships [36] and was associated with physical activity in older adults [37] and adolescents [38]. The evidence suggests that the first agents responsible for subject socialization, enabling the transmission of standards and values, are parents and siblings [35]. The social groups in which individuals are inserted (such as family) may influence their habits, as well as encourage them in practices of shared interests [39]. This is also indirectly reflected in sports, in which families that have a sports practice in common there may have a trend for athletes to be more involved in training, motivated by the desire to learn more about the practice and achieve sports excellence [35]. Through a higher training commitment (weekly volume, frequency), runners can experience performance improvement [40].

These results agree with our findings about the role of the runners in family members for men since the results also showed that family members practicing running were related to higher weekly volume, sport during childhood, and participation in a running group. The importance of family support for physical activity as well as for running commitment [15, 41] was previously mentioned, highlighting that coordination and cooperation between partners are important to organize the time for training commitment [41]. For the present study, sharing the same practice among family members could be related to higher indicators for training characteristics for men (40.7 vs. 26.9 km/week), compared to women.

The importance of sports in childhood for women was an interesting result, refuting our hypothesis about the role of family members in training engagement. These results suggest that past sports practice was the most important simple rule for connecting other variables in the network. However, the importance of family members for running practice should not be neglected, since our results also showed a positive association between sports during childhood and the presence of other runners in the family, as well as a positive association between runners in the family and training frequency, and running experience. Considering the design of the study, inferences about the mechanisms that explain this association are a challenge. However, considering the stability of physical activity, previous studies highlighted that physical activity during childhood is positively associated with physical activity in adulthood [42, 43]. Therefore, the present results suggest that running performance was positively related to involvement in sports during childhood, highlighting the relevance of incentive sports practice for girls. However, future studies need to consider the influence of sports during childhood to start into the running practice and characteristics of sports activities during childhood (i.e., practice time, team sports, individual sports) most important for running during adulthood.

Firstly, the limitations of the present study include the lack of information regarding civil status, which can impact runners’ motivation and modify social support results [44]. We showed the role of the family members for men; however, non-information is available regarding the characteristics of these relationships, if it is related to the partner, offspring, parents, siblings, or extended family. Secondly, training variables do not include the training methods and exercise intensity, which can present different outcomes for performance. Thirdly, information regarding environmental perception is lacking, which can impact the training commitment, especially for women [45]. Nonetheless, the present study advances the comprehensiveness regarding the relative importance of social support for runners' commitment and performance. These findings have practical applications for health policymakers. The incentive for sports practice during childhood should be considered, especially for girls since this variable presented a higher expected influence in women's network. In addition, the cost reduction for running events participation in family, as well as the inclusion of activities (for leisure, health, and well-being), engages family members of different age groups in running activities should be considered.

## Conclusion

Running engagement among Brazilian amateur runners was related to the existence of other runners in the family and past involvement in sports practice, for men and women, respectively. For training characteristics, the weekly volume was the most crucial variable for Brazilian runners’ performance. Results reinforce the role of training characteristics, as well as the importance of sports practice during childhood and running practice in the family.

## Data Availability

The datasets used and analyzed during the current study are available from the corresponding author on reasonable under request.
